# Illness Experiences of Adolescents with Type 1 Diabetes

**DOI:** 10.1155/2022/3117253

**Published:** 2022-12-20

**Authors:** Ji Eun Kim

**Affiliations:** Konyang University, 158, Gwanjeodong-ro, Seo-gu, Daejeon 35365, Republic of Korea

## Abstract

**Objective:**

This study is aimed at exploring the illness experiences of adolescents with type 1 diabetes (T1D).

**Methods:**

Using semistructured questions, 12 adolescents with T1D in Korea were interviewed regarding their illness experiences. Data were analyzed using grounded theory methodology.

**Results:**

Participants described their experiences in the core category, “becoming myself again,” which resulted in 130 concepts, 36 subcategories, 13 categories, and five themes. The themes included tied, overwhelmed, running away, struggling, and conciliating. Tied, the first process, entailed three categories: “confined to blood sugar control,” “feeling constrained,” and “supervised by parents.” The overwhelmed process included “self-banished” and “surrounded by stigma.” Running away included three categories—“growing up,” “folding,” and “withdrawal.” Struggling entailed “seeking for solution to overcome,” “developing response skills,” and “accepting help.” The last process, conciliating, included “redefining my perspective” and “reconciling with myself.”

**Conclusions:**

The findings indicate that the illness experience of adolescents with T1D should be understood in terms of both physical and psychosocial dimensions, considering the sociocultural and developmental context. The results of this study provide valuable information on diabetes education. Moreover, these findings encourage nurses to develop intervention programs and strategies to help adolescents with T1D.

## 1. Introduction

Type 1 diabetes (T1D) is the most common endocrine and metabolic disease in children and adolescents, and the main age of onset is generally less than 20 years [1]. In South Korea, the incidence and prevalence rates are highest among those aged 15-19, and those under 19 years account for 28.4% of the total among this age group [2]. The incidence of T1D among Korean adolescents is significantly increasing. For those under the age of 15, the annual increase was 5.6% between 1995 and 2014, which is twice as high as the global annual increase of 2 to 3% [2, 3].

T1D results from the autoimmune destruction of islet beta cells, leading to insulin deficiency and hypoglycemia [4]. Therefore, to manage T1D, blood sugar tests are performed along with insulin administration throughout life, and diet control and exercise therapy are combined [5, 6]. Insulin treatment must be performed several times a day and requires knowledge and skills to use a syringe, store insulin, and select an injection site [6]. Moreover, insulin treatment causes a side effect of hypoglycemia [5]. However, insulin treatment with these efforts does not guarantee stable blood sugar in adolescence because increased hormone secretion and body growth affect the effect of insulin. Furthermore, the loss of beta cell function is in progress, so blood sugar changes are expensive [5, 7]. Adolescents manage diabetes at school alone unlike others their age and without parental help unlike children [8].

Adolescence is a period of flurry and developing self-identity; while experiencing growth amid major physical and mental changes and reestablishing social roles, it is easy to express unstable emotions amid this chaos [9]. The fact that adolescents' treatment attitudes for T1D are resistant along with the characteristics of these stages of growth and development is distinctly different from that of children's cooperation [10, 11]. Insincere treatment adherence in adolescents with T1D causes psychological problems and conflicts with their parents [11–13]. These social and psychological difficulties lead to a vicious cycle that leads to a lack of treatment adherence and negatively affects the results of diabetes management [13].

Accordingly, previous studies on adolescents with T1D have focused on concepts related to knowledge and skills, such as insulin administration and nutrition, to promote diabetes management [14, 15]; coexisting mental health diseases, such as depression and eating disorders; and social aspects of problematic behaviors, such as aggression and hostile defiance, as well as school life adaptation and peer relationships [15]. Intervention studies based on these concepts have been conducted from various angles at the individual and group levels [14, 15], but according to a systematic literature review on program studies for adolescents with T1D, adolescent diabetes programs have low levels of effectiveness. A study showing relatively high effectiveness was regarding a program in which several concepts were combined [16]. In other words, diabetes management in adolescents with T1D requires a systematic and comprehensive understanding rather than a partial approach. As such, most previous studies are insufficient to provide an in-depth understanding of the effects of T1D on life and management experience.

Qualitative research on adolescents with T1D has rarely been conducted abroad, and it focuses on a selective subarea [10, 17, 18] or includes children and parents as subjects [17, 19]. Thus, it is difficult to understand the entire process of adolescent illness, and there are limits to understanding it within the sociocultural context of Korea. The basic premise of grounded theory is that human beings create awareness of themselves and objects based on symbolic interactions with society [20, 21]. Thus, grounded theory methodologies enable us to understand how people with illnesses give meaning to the illness itself and the experience of living with it in a social context [22]. Therefore, the purpose of this study was to develop a substantive theory that can explain the experience process by identifying the meaning that adolescents with T1D give to their illness experiences and the social context that causes them. The research question of this study was as follows: “What is the illness experience of adolescents with T1D?”

## 2. Methods

### 2.1. Setting

This qualitative study utilized Corbin and Strauss' grounded theory methodology to understand the illness experiences of adolescents with T1D [20, 21]. Since grounded theory has an epistemology that respects the expertise of human beings who have experienced the phenomenon [23], researchers should be able to understand the shared meaning of participants and cultural basis of the communication expressed well. In this regard, I had worked as a school nurse at secondary school public health teacher for more than 10 years and is skilled in communicating with them. Therefore, this researcher was expert in sociopsychological development and social interaction of adolescents and prepared to understand the lives of participants.

The Institutional Review Boards of Anam and Kuro Hospitals of Korea University Medicine approved this study. Before commencing the study, the researcher explained the purpose of the research and participants' right to the participants and their parents. Informed consent was signed by all participants and their parents with their assurance of their confidentiality and withdrawal right to the interview at any time.

### 2.2. Participants

Purposive and theoretical sampling were used to recruit a sample of 12 adolescents with T1D diagnosed more than 1 year and age 13-19. I recruited from the Department of Pediatrics at University Hospitals and through the introduction of participants.

Of the 12 adolescents, 58.3% were female, 41.7% had the duration of illness ranged from 2 to 4 years, and 58.3% uncovered their illness at school (see Table 1). Regarding the insulin administration method, all participants used the injection type, and there were no pump-type users.

### 2.3. Data Collection

An in-depth interview was conducted with all participants that used open-ended and semistructured questions. The face-to-face interviews lasted from 40 min to 2 hours and 15 min for each participant and were conducted 1-2 times per person within 12 months. The interview location was selected considering participants' convenience and psychological stability. All interviews were audio-recorded and transcribed verbatim including the nonverbal part and time taken and were used as the raw material.

Relatively, young adolescents find it difficult to answer open-ended questions with high abstraction; therefore, the method of presenting the questions through analogies or examples was used. As the interviewee was a student and called the researcher “a teacher” in Korean, the researcher used the honorific so as not to confuse the interview attitude. Therefore, at the beginning of the interview, rapport was formed through routine questions that were not judged based on one's own answers. Afterward, the interview began with general questions, such as “When and how did you get diagnosed with T1D?” Specific questions included “Tell me about what you experienced or felt while living with T1D,” “How are you adjusting to T1D?”, and “What is needed for adolescents with T1D? What do you think?”

After the initial interview, outline concepts were derived through open coding, their categories were constructed, and the categories were reviewed. Thereafter, the concept derivation was comparatively analyzed and revised at each interview; when no new categories appeared during the interview, it was considered saturated.

### 2.4. Data Analysis

The analysis simultaneously began with data collection, taking notes of ideas during on-site interviews, transcriptions, and concept coding and drew up a chart to organize the relevance of emerging concepts and categories.

First, to perform open coding, all transcripts were coded for each sentence in a word program. Thereafter, the coded concepts were sorted by level between concepts and categories using Excel. Subsequently, they were comparatively analyzed to extract concepts through similarities and differences and categorize them while integrating similar concepts. In the process analysis, first, the categorized content was abstracted from a symbolic and interactive point of view and labelled as themes. All the analysis processes were analyzed iteratively and cyclically in every interview and step, so that the emergence of concepts was saturated. Ultimately, I presented the theory to be refined as presenting the action interaction in the whole context that could confirm symbolic interaction, which is the basis of grounded theory.

## 3. Results

**Figure 1:**
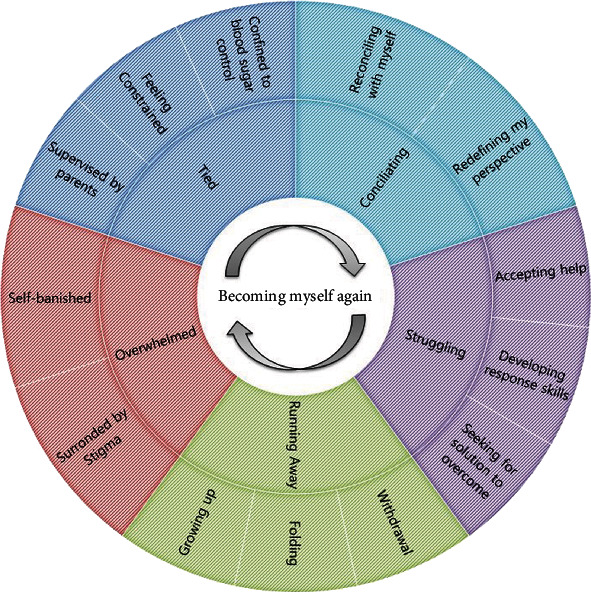
Process model of disease experiences of adolescents with type 1 diabetes.

As a result of analyzing the data on the illness experience of adolescents with T1D, 130 concepts were derived in the open coding stage and abstracted into 36 subcategories, 13 categories, and five themes (Table 2, Figure 1). Pseudonyms were used to protect the participants' anonymity.

### 3.1. Tied

The tied process included confined to blood sugar control, feeling constrained, and parents' supervision. Participants felt constrained by diabetes management because they had to be managed accordingly. And the vicious cycle in which symptom management leads to additional management aggravated the phenomenon of being caught in the management of blood sugar levels. For example,

low blood is bad. Low blood flow, you will then be hungry. You eat far more than you should. After eating, you have to get an injection. (Mia)

Feeling constrained derived from diet the most regardless of their big appetite or hunger. Uncontrolled blood sugar interfered with physical activity or sleep and is perceived as a limitation in daily life, as well as in academic and career settings. For example,

controlling what I eat is the most difficult task. In high school, my classmates went in and out of the canteen so often. I mean, they are full of the smell of bread and ramen. I thought I was going to die of starvation. (Gray)

Parents' supervision is attached to difficult T1D management. Participants depended on their parents and followed their instructions. Parental concerns rationalized excessive intervention and transformed diabetes management into a test. Examples follow:

Daddy has his own way. If I do not measure it, he gives me a blood glucose meter. If I sleep, then my dad measures me. My dad used to say, “Just listen to what I say.” I think Dad might worry about me. But actually self-satisfaction. (Steve)

Mom looking at the result of high glycated hemoglobin “let us go exercise immediately.” So, I woke up at 5 am and went swimming during vacation. Before (blood sugar) went over 200, I used to go out to exercise and take measurements. Because my mom was horrified. (Mia)

### 3.2. Overwhelmed

The overwhelmed process included self-banished and surrounded by stigma. Participants experienced the expulsion and banishment of their original selves. The existing self with health was expelled by overlaying their identity with diabetes. They recognized that diabetes made them different from their peers and limited their lives and functions as a flaw that drove them to humiliation. For example,

I thought this was my one flaw. I should be a straight person without blemishes. Therefore, my scratches. “Oh, I have to be perfect,” I think that made me even more depressed. (Amy)

Participants were surrounded by stigma; prejudice and discrimination were hidden in the form of sympathy and caring. All participants complained about social prejudice, whereby they were blamed for the cause of T1D as being due to their wrong lifestyle. While curiosity about illness management lasted for a certain period, discrimination, such as sympathy or alienation, was no deadline, thus making participants reluctant to disclose their diabetes. Examples follow:

(Crying) I think a certain boy made fun of me (crying for about two minutes) calling me “patient.” I did not say anything. Didn't think of anything. Shocked. It is a disease. (Continues crying) From then on... I did not want to reveal it. I was just embarrassed. (Michelle)

It's not my fault (crying) “She's obese, she ate a lot.” I did not do anything and I got caught. (Margot)

### 3.3. Running Away

The running away process included growing up, withdrawal, and folding. It developed as an interaction to protect the self from previous processes. Growing up was a pivotal part of this process and separates the experiences of illness in adolescence from those of childhood. Participants went through a time of wandering about themselves during puberty, which negatively affected diabetes management. They criticized and re-recognized the diabetes knowledge and management rules that were instilled in childhood. While peers and parents grew up together, their understanding was broadened, and parental coercion was decreased. Examples follow:

Little by little from my first grade in middle school I did not manage diabetes. But l tended to do it more than in my second year (laugh). (Michelle)

I ate a lot in middle school. However, these days, strangely, I do not think about eating. (Omitted) It takes time, and a lot of time to get used to it. Time is of essence, and diabetes is no exception. It takes time to adapt and accept it. (Tiffany)

Folding means trying to escape influence by avoiding and denying the operation of the external system surrounding the self. Direct resistance to treatment was conspicuous in 8th grade syndrome. Neglecting was a comparatively avoidant and passive resistance. It was participants' reaction to illness itself and their parents' coercion. For example,

when I got drinks from my parents because of my low blood sugar, I spilled and did not drink, “I will die, and I will not eat something like this.” (Amy)

Withdrawal is, nonetheless, an attempt to protect the self through isolation and concealment from others. Participants' perceived concealment was necessary by experiencing the negative reactions of others to diabetes or by listening to the indirect experiences of patients. For example,

(during my school trip) I kept walking. It continues to drop (blood sugar level). I kept drinking juice, and I do not know why my blood sugar level was 56. I do not know whether I slept or fell faint. When I woke up and found that I was alone lying in my (dorm) room. My friends go out (without knowing my condition). (Peter)

### 3.4. Struggling

The struggling process was achieved by actively interacting of the adolescent with the external system, such as seeking solutions, developing response skills, and accepting help. Therefore, this process acted antagonistically on the negative aspects of the previous processes, thus reducing those influence. Participants tried to carry out their own thoughts on adherence to treatment. Seeking solutions to overcome has led to an attempt to no longer allow diabetes to be perceived by self and others as a fault. Accordingly, they revealed diabetes to their peers first and appeared to supplement the self that was reduced by diabetes. Examples follow:

I think it is okay to do it my way. This is the right way for me. Whatever they say is their own opinion. If you believe in your own experience and find it difficult to be stereotyped a bit, living like that is not bad. (Steve)

All children in my class know that I am diabetic. I told all students standing in front of the school table. I did not want to hide it. (GD)

Participants developed their own management knowledge and skills as the disease period elapsed regardless of their attitude toward acceptance. They also became accustomed to coping with the stigma. They utilized a method of dispersing gaze to prevent stigma or representing weaker reaction to reduce the intensity of the teasing-like humor. For example,

I almost know it now. When I was young, I did not know about self-injection, and blood sugar check. Now I know basic common sense. Technical part and dietary part. (Peter)

I took it in a very uninteresting way. In fact, when someone bullies you, when you react to it, the children actually do it because it is more fun. Just “Are you on drugs?” Then it's like, “Oh, I am on drugs. Would you like to join us?” If this is the case, it will pass, no matter what. (Gray)

Participants accepted practical help from their parents and peers regarding diabetes management. Parents supported all areas of diabetes management and conveyed positive aspects of disease awareness. Participants also experienced support of school members in responding to stigma. Moreover, group activities for patients with T1D relieved the sense of isolation and provided empathic support for participants. For example,

I carefully talked to two of my friends, and they helped me a lot. So, I think I'm more comfortable accepting them than the first time, because they approached me first and told me to go to the bathroom at lunchtime (to manage my diabetes). (Emma)

### 3.5. Conciliating

The last process, conciliating, included redefining my perspective and reconciling with myself. Participants perceived diabetes with their own knowledge, which contradicted the previously implanted perception. They evaluated the benefits and the burden of symptoms and coping with diabetes. They recognized their illness as acceptable and acknowledged it not avoidable. This was not a defeatist and passive interpretation but an approval. They set actionable management goals and played the role of the main agents of treatment adherence. Examples follow:

I am not actually lying; because of this illness, I have benefited more. I already know what you eat (blood sugar) goes up. If I want to go on a diet (laughs), I think it will be easier for me to become healthy than others and to improve my body more easily. (Katy)

I have no choice. Most people do not want to accept it. Some people give up. But I just do. And if I am going, I do it anyway. How can I not do it? I cannot help it (diabetes). (GD)

Reconciliation with myself began with acceptance of illness. Temporary acceptance was approved until participants reached a tolerable range or were completely cured, so they wanted to get out of management as much as possible. However, gradual acceptance was based on participants' positive evaluation of their health status, even after years of morbidity. They gave up hope for a cure, accepted diabetes for what it was, and considered realistic and long-term management. Examples follow:

I was talking about surgery. I think it will not fail and will be possible when I am in college. First, if you do not get complications right now, you are going to have the surgery, and you are already blind and your legs are rotting, so what's the use? That makes me manage diabetes for healthy surgery. (Tiffany)

I do not think it will be cured in my life. I hope there'll be product development, though I do not want to be cured. (Katy)

After being diagnosed, I slightly changed my mind. This gave me an opportunity to change. My puberty went without a hitch. If you have diabetes and take good care of your mental health, you will not experience any problems. (Tom)

## 4. Discussion

The core category of the illness experiences of adolescents with T1D in this study was “becoming myself again.” The emergent themes of tied, overwhelmed, running away, struggling, and conciliating interact and explain the process. Mead [24], who proposed symbolic interaction theory, said that the subjective response of the self can construct and change meaning through interaction with groups and systems. This study confirmed how adolescents construct the meaning of T1D and management in their lives as they interact their awareness with others within the social context.

Participants experienced “becoming myself again” by acting and interacting from the self-banished to the acceptance of diabetes. Previous studies are consistent with the view that adolescents' chronic illness experiences are “getting out of the withered self” [25] and “accepting the self by overcoming an unstable sense of self-control” [26]. This is in stark contrast to the central phenomena of physical pain [27, 28] and social identity crisis [29] in adults with chronic illness. As such, it is uniquely shown in research on adolescents that self is threatened by chronic illness and then realigned. The “becoming myself again” concept is consistent with the results of previous studies [30, 31] that individuals reconstruct their own interactions, emotions, and definitions of illness, which lead to changes in the self.

In the first process, tied, participants felt caught in the restrictions due to the control of blood sugar, transferred the sovereignty of diabetes management to their parents. For participants, diabetes management was an endless task that had to be solved by depending on the symptoms of hypoglycemia and hyperglycemia, and participants had to endure inconveniences, such as securing time, moving places, and carrying items. It is consistent with the study by O'Hara et al. [32] who found that diabetes management was a cumbersome task for people with T1D. Furthermore, it is in line with the findings of McDonough et al. [33] and Scholes et al. [18] that adolescents recognize they are controlled by their blood sugar control. Previous studies, which concluded that diabetes management itself brings blame and nagging to parents for adolescents with T1D [12, 34], support the result that participants were evaluated by their parents based on blood sugar level and were bound to concern. Therefore, physical and environmental approaches, such as securing convenience through the use of devices and preparing conditions for diabetes management in the classroom, should be considered to minimize the degree to which adolescents with T1D feel obsessed with diabetes management.

In the next process, overwhelmed, participants recognized the pressures of T1D that banished the self. They perceived the illness according to the embodiment of living with T1D, since the body becomes the basis of the self through embodiment [35]. They confirmed that their forced identify, diabetic, was different from their peers. It is consistent with the previous studies suggesting that this sense of heterogeneity made adolescents being aware of them outside the normal range [8, 17].

The stigma that participants experienced encompassed their whole lives in various forms, such as questioning, sympathy, rebuking, and consideration. Stigma brought participants a sense of humiliation which they were unable to confront. It is consistent with Sausse's [36] report on stigma, which states that disabled people are unable to respond to the insults and exclusion of others and become silent. Furthermore, Leidner et al. [37] stated that humiliation is a feeling of helplessness and high anger. Previous studies have shown that humiliation is caused by the perpetrator to the victim in a state of imbalance of power [38] and that adolescents with T1D are greatly influenced by their peers' responses to the illness at school. This finding suggests that school-level illness awareness education is necessary.

Participants showed dramatic changes in their attitudes toward T1D starting with the onset of puberty, and this growth was the basis for the running away process. Previous studies have shown that adolescents with T1D no longer have the adaptive attitude of childhood, such as experiencing conflicts with parents without exception [11, 39]. However, in the second half of adolescence, as they grow up, problem behaviors decrease [40] and conflicts with parents are almost resolved [39]. This supports the result that participants experienced fewer conflicts with their parents whose anxiety were decreased and experienced a greater understanding of their peers, when they entered high school at the age of 16. Therefore, interventions for emotional support and transition education according to the growth should be studied in the future.

Folding involves denial and avoidance, in which the self with growth and strength tries to run away in response to the previous process. Participants showed willful negligence in adherence or refused to accept the illness. However, this representation of maladaptation was the coexisting opposite aspect of the adaptation mechanism that reduced psychological shock and burden. Previous studies on chronic illness have also viewed denial as a defense mechanism in the problem of illness acceptance as a strategic adaptation to chronic illness [27, 40, 41]. Most participants felt withdrawn as they accelerated concealment and isolated themselves that they had to do it alone. Previous studies announced that the stigma by peers made illness concealed, which made it more socially withdrawn [17, 34]. Thus, rapid attitude change toward illness of growing adolescents needs to be understood as a characteristic of the running away, not a problem with ostensible rebellion for adherence.

Adolescents experienced the struggling process of finding ways to recognize and manage T1D themselves as the subject of the illness. Schulman-Green et al. [42] stated that living with a chronic illness means taking ownership of health needs; participants tried to live with diabetes in their own way as subjects of diabetes against others and society who made them recognize diabetes as a defect. Moreover, participants pursued alternative ways to overcome diabetes. This is consistent with the compensatory theory that appears universally in chronic illness adaptation [43]. Participants even preemptively disclosed the presence of diabetes. This is in line with the protective disclosure mentioned by Charmaz [44] to control the negative consequences of unintentional exposure, setting the perception of the illness as a defect.

Developed response skills weakened the overwhelmed and accelerated the conciliating, even if it was improved involuntarily by parental coercion. Previous studies mentioned that self-care for chronic illness starts with discerning the symptoms of the body and thus adolescents with T1D should learn through trial and error for their illness adaptation and independence achievement [19, 42]. Accepting help from important others induced positive management results, as parents provided an environment in which adolescents could acquire diabetes knowledge and skills [19]. Participants acknowledged that parents continued their auxiliary role as supporters, which is consistent with the argument of previous studies that even a simple form of support is important as an effect of continuous parental support [40]. In a previous study, the emotional and intellectual help that participants received from other patients with T1D could exchange therapeutic values and reduce the burden of diabetes management and improve diabetes management behavior [45]. Therefore, health providers should be able to provide adolescents a safe environment for various attempts to recognize and manage symptoms of T1D.

In the final process, conciliating, participants became reaware that diabetes can be an advantage, not necessarily a drawback, to their health. Acknowledging the inevitability of acceptance achieved reawareness of the illness and promoted the integration of diabetes into one's life, personalizing the goals of diabetes management, enabling proactive adherence, and reconciling with the self. O'Hara et al. [32] reported that adapting to diabetes is to redefine the relationship with diabetes by reducing the severity of diabetes with a change in perception. Wright and Kirby [46] argued that a new cognitive approach to existence and life should be applied by pursuing personalized goals in treatment adherence.

Participants built their own principles for treatment through preceding process. As such, the conciliating is related to the fact that illness experience balances daily life with illness and a new self [43, 47].

The conciliating started with “reconciliation with myself.” Wright and Kirby [46] divided the acceptance of diabetes into active acceptance, to integrate the illness with life, and resigned acceptance, in which the integrity of life is cut off because of the illness. They stated that the degree of illness management depended on this mutually exclusive form of acceptance. In this study, it can be said that the subcategory of “not making diabetes a fault” in the struggling corresponds to active acceptance, and the subcategory of “acknowledging the inevitability of acceptance” in the conciliating corresponds to the form of resignation acceptance. There were many participants holding two attitudes concurrently. If the inevitability of acceptance was acknowledged, the necessity of diabetes management was acknowledged, which leads to a high degree of treatment adherence. Although it was a resigned acceptance, the illness did not cut off the integrity of life. Acknowledging the inevitability of acceptance enables the recognition of diabetes characteristics and gradual acceptance. Furthermore, it facilitated the integration of diabetic illness into one's life, enabling approved constancy of self and significantly reducing the interaction of running away. Further research is needed to determine whether this is due to cultural differences in attitudes toward accepting chronic illnesses. Therefore, nurses must provide interventions that respect and support the patient's experience without evaluating the type of acceptance and individual recognition of adolescents with T1D on a uniform basis.

There are several limitations of the current study. Primary among these is that the data were limited in participants' experiences. Reachable physiological data like HbA1c or blood sugar level were obtained exclusively by self-report. And some participants did not check or remember their blood sugar levels. Moreover, some participants negated the HbA1c results by inducing continuous hypoglycemia. Above all things, participants' responses to diabetes-related numbers were defensive for they were sensitive to being judged. Although I cannot completely address the interpretation of adolescents' experiences related to their physiological health status, I focused on their subjective expression, emotions, and opinion excluding researchers' prejudice.

A second limitation is that the contents of experiences are vast. Although participants are young, their experiences of living with T1D are not limited to disease management but are very diverse across all areas of life. The specific aspects shown as themes in this study must be identified concentrically, especially those that have not been explored in previous studies with a qualitative approach. Nonetheless, the present findings show holistic insights into the life of adolescents with T1D.

A third limitation is that our small and ethnically homogeneous samples limited exploration of the trends beyond the culture such as parental discipline style, parent-child communication, and preference for technology. However, the value of qualitative research is informative; therefore, it does not pursue generalization with representative samples for analysis [48].

## 5. Conclusion

This study attempted to provide an in-depth understanding of the meaning and dynamics of illness and management of adolescents with T1D in the interaction between adolescents and the society surrounding them using a grounded theory methodology. As a result, I developed a substantive theory about the illness experience of adolescents with T1D which compose a dynamic process of five themes. This provides the basis for nursing interventions for self-initiated health management of adolescents with T1D in the future. However, since it is a study that examines the accumulation of experiences from a single point of view, the approach is limited according to the individual characteristics of the subjects.

I suggest future research that can provide optimal nursing interventions and education for adolescents with T1D by categorizing them related to their developmental characteristics based on an integrated perspective across all areas of life. Furthermore, it is necessary to help adolescents with T1D to experience the illness on their own by including parents, school members, medical personnel, and patient groups who surround adolescents in nursing education.

## Figures and Tables

**Table 1:** Participant demographics.

Demographic categories	Frequency	Percentage
Gender		
Male	5	41.7%
Female	7	58.3%

Age (years)		
13-15	2	16.7%
15-17	6	50.0%
17-20	4	33.3%

Duration of illness (years)		
2-4	5	41.7%
4-6	2	16.7%
6-8	3	25.0%
8-11	2	16.7%

Age at the time of onset (years)		
5-7	2	16.7%
7-13	7	58.3%
13-16	3	25.0%

**Table 2:** Themes, categories, and subcategories.

Themes	Categories	Subcategories
Tied	Confined to blood sugar control	Responding to symptoms according to changes in blood sugar
		Troublesome
		Exhaustion
	Feeling constrained	Limited food consumption
		Limited daily life
		Limited social activity
	Supervised by parents	Dependent on parents
		Parental coercion on management
		Excessive concerns from parents
		Being judged by parents

Overwhelmed	Self-banished	Defined as a diabetic
		Alienated from others
		Humiliation
		Despair
	Surrounded by stigma	Information delivered unilaterally
		Alerted from the possibility of stigma

Running away	Growing up	Psychological development
		Growing up with others
	Folding	Resisting diabetes management
		Pretending not to be diabetic
	Withdrawal	Concealing
		Isolated

Struggling	Seeking for solution to overcome	Becoming the subject of management
		Not making diabetes a fault
	Developing response skills	Coping with stigma
		Improving management skills
	Accepting help	Blood sugar management support
		Emotional support
		Support against stigma

Conciliating	Redefining my perspective	Acknowledging the benefits of diabetes
		Surmountable
		Acknowledging the need T1D management
		Acknowledging the inevitability of acceptance
		Setting actionable management goals
	Reconciling with myself	Temporary acceptance
		Gradual acceptance
		Approved constancy of mine

## Data Availability

The data that support the findings of this study are available on request from the corresponding author. The data are not publicly available due to privacy or ethical restrictions.
